# Online updating of obstacle positions when intercepting a virtual target

**DOI:** 10.1007/s00221-023-06634-5

**Published:** 2023-05-27

**Authors:** Emily M. Crowe, Jeroen B. J. Smeets, Eli Brenner

**Affiliations:** 1 Department of Human Movement Sciences, Institute of Brain and Behavior Amsterdam, Amsterdam Movement Sciences, Vrije Universiteit Amsterdam, 1081 BT Amsterdam, The Netherlands; 2 School of Psychology, University of Nottingham, University Park, Nottingham, NG7 2RD UK

**Keywords:** Online control, Interception, Perturbation, Adjustment, Obstacles

## Abstract

People rely upon sensory information in the environment to guide their actions. Ongoing goal-directed arm movements are constantly adjusted to the latest estimate of both the target and hand’s positions. Does the continuous guidance of ongoing arm movements also consider the latest visual information of the position of obstacles in the surrounding? To find out, we asked participants to slide their finger across a screen to intercept a laterally moving virtual target while moving through a gap that was created by two virtual circular obstacles. At a fixed time during each trial, the target suddenly jumped slightly laterally while still continuing to move. In half the trials, the size of the gap changed at the same moment as the target jumped. As expected, participants adjusted their movements in response to the target jump. Importantly, the magnitude of this response depended on the new size of the gap. If participants were told that the circles were irrelevant, changing the gap between them had no effect on the responses. This shows that obstacles’ instantaneous positions can be considered when visually guiding goal-directed movements.

## Introduction

We often make reaching and grasping movements in cluttered environments consisting of both target and non-target items. The presence of non-target items in the surrounding alters the spatial trajectories of both reach-to-grasp (e.g. Howard and Tipper 1997; Jackson et al. 1995; Tresilian 1998; Mon-Williams et al. 2001; de Grave et al. 2005; Saling et al. 1998; Menger et al. 2012; Tresilian et al. 2005; Kritikos et al. 2000; Verheij et al. 2014; Voudouris et al. 2012) and pointing movements (e.g. Brenner and Smeets 1997; Chapman and Goodale 2008; Chapman and Goodale 2010a; Brenner and Smeets 2007; Tresilian 1998; Dean and Bruwer 1994; Sabes and Jordan 1997). This shows that the positions of non-target items are evaluated and incorporated into the planning of arm movements, presumably to avoid unwanted collisions.

Since neither one’s own movements nor the behaviour of objects in the environment are completely predictable, it is important to be able to adjust ongoing reaching and grasping movements. We know that people make fast, automatic adjustments to account for changes in a target’s position (e.g. Brenner and Smeets 1997; Brenner and Smeets 2003a; Day and Lyon 2000; Oostwoud Wijdenes et al. 2011; Prablanc and Martin 1992; Soechting and Lacquantiti 1983; Veerman et al. 2008). We also know that they respond quickly when the visually perceived position of a representation of their hand (Saunders and Knill 2003; Sarlegna et al. 2003) or of a tool that is guided by their hand (Brenner and Smeets 2003b) is suddenly displaced. This shows that the perceived position of both the target and hand are continuously used to adjust ongoing arm movements (reviewed by Brenner and Smeets 2018).

Non-target items in the environment can also change position during reaching movements. Are positions of such items also continuously updated, allowing for fast adjustments of the ongoing movement? Aivar et al. (2008) found that participants responded to sudden displacements of virtual obstacles positioned between the start and endpoint of their movement. However, the (initial) response followed the retinal motion signals induced by the obstacle displacements rather than being an adequate adjustment to the constraints imposed by the new positions of the obstacles. Thus, this response to a change in obstacle position might not be the result of continuously updating their positions but may instead be a response to motion near the movement endpoint (e.g. Crowe et al. 2021; Crowe et al. 2022). Moreover, the responses to changes in the position of two obstacles defining a gap has a longer latency than the response to a change in the position of an equally large pass-through target (Aivar et al. 2015), suggesting that obstacles and targets are considered differently.

Thus, we know that one can respond to a change in obstacle position, but that this response differs from that to a change in target position. Does one consider the most recent information about obstacles when one responds to a change in target position? Chapman and Goodale (2010b) asked participants to make reaching movements towards a target that sometimes jumped to a new position during their reach. For the new target position, physical items that were initially irrelevant for the reach sometimes became an obstacle. When this happened, the reach trajectories to the new target location were affected, showing that obstacle positions are considered when adjusting ongoing arm movements. However, the question remains whether only obstacles’ positions when the movement is planned are considered when adjusting the movements, or whether obstacles’ positions are constantly updated such that their locations at the moment of the adjustment are considered.

We used a virtual environment to find out whether the adjustment to an ongoing arm movement in response to a target jump considers the latest estimate of the positions of obstacles in the surrounding. We asked participants to slide their finger through a gap between two circular items. Half of the participants were told these items were obstacles and should be avoided, whereas the other half were told they were irrelevant. The reason for including irrelevant items was to check for direct responses to the obstacles’ motion because motion of irrelevant items can influence the finger’s path (e.g. Crowe et al. 2021; Crowe et al. 2022). We designed the task such that we do not expect systematic responses to the items’ motion when the gap changes size because the two identical items moved by the same amount in opposite directions. At a fixed time during each trial, the target suddenly jumped slightly along its path whilst continuing to move. We expected the finger to respond to such jumps, because this has frequently been demonstrated to occur (e.g. Brenner and Smeets 2015; Brenner et al. 2022). The gap between the items also changed size on every trial. This either happened when the target appeared (i.e. ‘early’) or at the time of the target jump (i.e. ‘late’). The goal of having the obstacles already change position when the target appeared was to check that the chosen obstacle positions influence the response to the target jump if they are already known when the movement is planned (as in Chapman and Goodale 2010b; Nashed et al. 2012). If participants consider obstacles’ instantaneous positions, then the response to the target jump should be tailored to the new gap size, even if the gap changes size at the same moment as the target jump. Thus, our main question is whether the response to the target jump is modulated by the gap size when the gap size changes at the same moment as the target jumps.

## Main Experiment

### Participants

Twenty-four participants took part in the experiment. Participants either volunteered to take part or took part in return for course credit. Twelve participants (all right-handed; 28 ± 5 years; we report values as mean ± standard deviation throughout this paper) were randomly assigned to the *obstacles* group and 12 (all right-handed; 26 ± 6 years) were randomly assigned to the *irrelevant item* group. Despite the cost in statistical power, we used a between-groups design to make it easier for participants to follow the instructions regarding the nature of the items. The study was approved by the local ethics committee in accordance with the Declaration of Helsinki.

### Setup

The experiment was conducted in a normally illuminated room. The stimuli were back-projected at 120 Hz with a resolution of 800 × 600 pixels onto a 1.25 × 1.0-m acrylic rear-projection screen (Techplex 15, Stewart Filmscreen Corporation, Torrance, California, USA) tilted backward by 30°. Participants stood in front of the screen. An infrared camera (Optotrak 3020, Northern Digital) that was placed at about shoulder height to the left of the screen measured the position of a marker (an infrared light emitting diode) attached to the nail of the index finger of the participant’s dominant hand at 500 Hz.

**Fig. 1:**
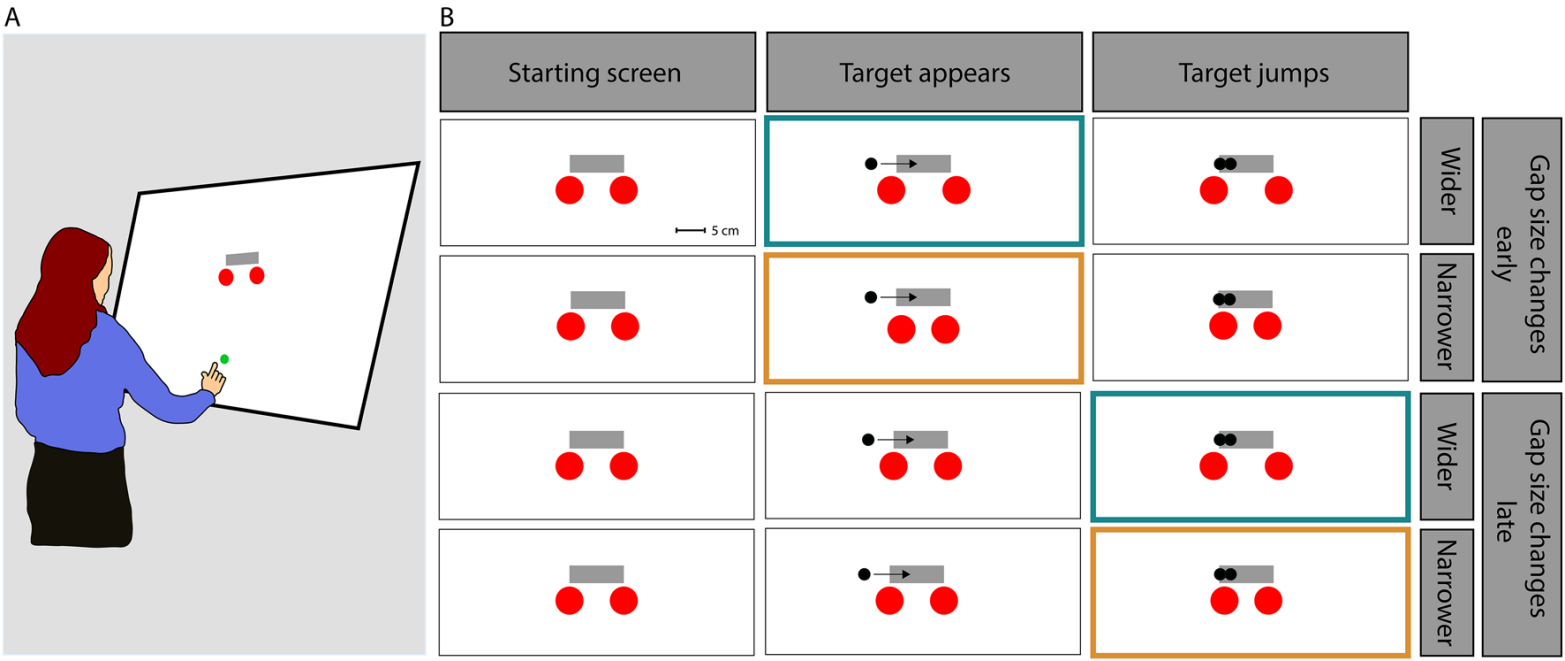
**A** Participant stood in front of the screen on which they perform the task. **B** The three main stages along the timeline of the stimuli. Each trial started with a grey hitting zone being presented above two red items with a gap between them (*Starting screen*). The target then appeared, immediately moving rightward across the screen (*Target appears*). Exactly 300 ms later, the target jumped either leftward or rightward (both options are shown but there was only one target; *Target jumps*). On every trial, the size of the gap between the items changed: it became either narrower (gold boxes) or wider (turquoise boxes). This either occurred at the same time as the target appeared (early) or at the same time as when the target jumped (late)

In order to synchronise the movement data (i.e. the marker position) with the stimulus presentation, the camera also recorded the position of a second marker attached to the side of the screen. This marker did not move but it stopped emitting infrared light so that its position was registered as ‘missing’ when a flash was presented at the top left corner of the screen (where a light-sensor was placed to detect the flash). We used a simple four-point calibration to relate the position of the fingertip to the projected images, automatically correcting for the fact that the marker was attached to the nail rather than the tip of the finger (Fig. 1).

### Stimulus and procedure

Participants stood in front of the large screen and were free to move as they wished. The display consisted of four virtual items: a black target (2 cm diameter disc), a grey hitting zone (10 × 3 cm horizontal rectangle, presented 15 cm above screen centre) and two red items (5 cm diameter discs), separated by a gap.

**Fig. 2:**
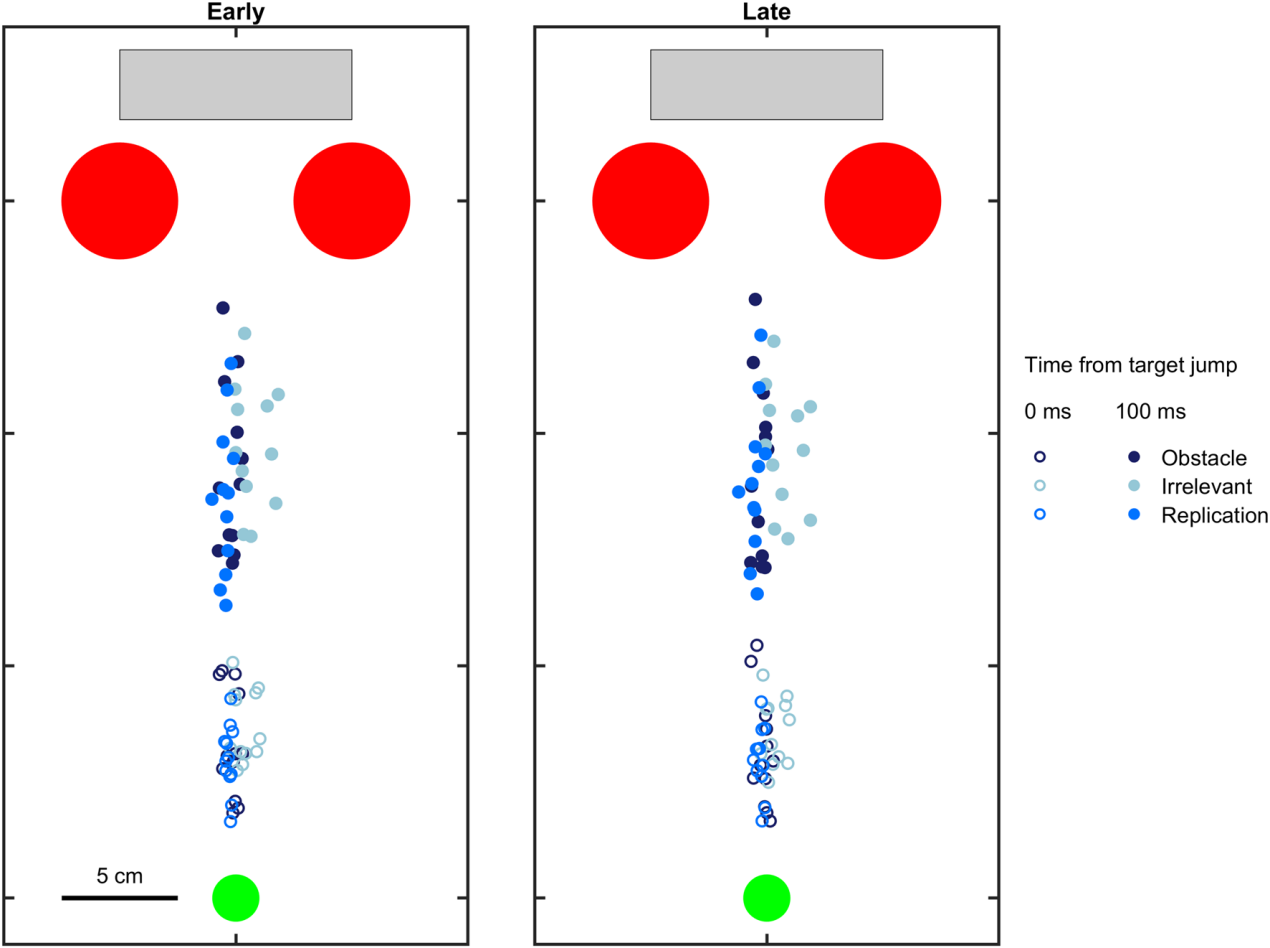
Position of the hand at the time of the target jump (empty circles) and 100 ms later (filled circles). Each circle shows the average position of the hand for an individual participant. Different colours indicate the three groups of participants. The left panel shows the position of the hand when the gap size changed at the time of the target appearance (i.e. early); the right panel shows the position of the hand when the gap size changed at the time of the target jump (i.e. late)

At the beginning of each trial, a green starting point (2 cm diameter disc) was presented 35 cm below the centre of the hitting zone. The gap between the red items was 5 cm wide and its centre was 5 cm below the centre of the hitting zone. To start a trial, participants placed the index finger of their dominant hand on the starting point. Between 500 and 700 ms after they did so, the starting point disappeared, and the black target appeared 10 cm to the left of the centre of the hitting zone, moving rightward at 20 cm/s. Exactly 300 ms after the target appeared, it jumped either leftward or rightward by 1 cm. We picked this time because it allowed participants to initiate their movement towards the target before the jump, but not be so close to the red items that they did not have time to respond. Figure 2 shows that all participants had left the starting point at the time of the target jump (empty circles) and had not reached the obstacles 100 ms later (filled circles), which is the earliest time we would expect them to start responding. The gap size also changed during each trial. It either changed when the target appeared (i.e. early) or when the target jumped (i.e. late). The gap size was either reduced to 3 cm (narrower) or increased to 7 cm (wider).

On arrival, participants read written instructions. These instructions were then repeated verbally to ensure understanding. Specifically, participants were instructed to slide their finger through the target whilst it was within the hitting zone. Participants in the obstacles group were told to avoid the red items, whereas participants in the irrelevant item group were told that the red items were irrelevant to the task. At that moment, participants were not aware that there were different groups who received different instructions. All participants were told that every time they successfully followed the instruction, they would receive *positive* auditory feedback and their score would increase by one point. If participants missed the target or hit it whilst it was outside of the hitting zone, they did not receive any auditory feedback. If participants in the obstacles group touched one of the red items, one point was deducted from their score and they received *negative* auditory feedback, irrespective of hitting the target or not. Within each group, all participants were subject to two timings of item movement (early; late) and two changes in gap size (wider gap; narrower gap). Each participant completed one block of 400 trials (100 trials for each of the conditions) in a single session that took approximately 20 min. In the 100 trials of each condition, the target jumped to the left on 50 trials and to the right on the other 50 trials. All conditions were randomly interleaved. At the end of the experiment, participants were debriefed, including being told about the groups.

### Data analysis

**Fig. 3:**
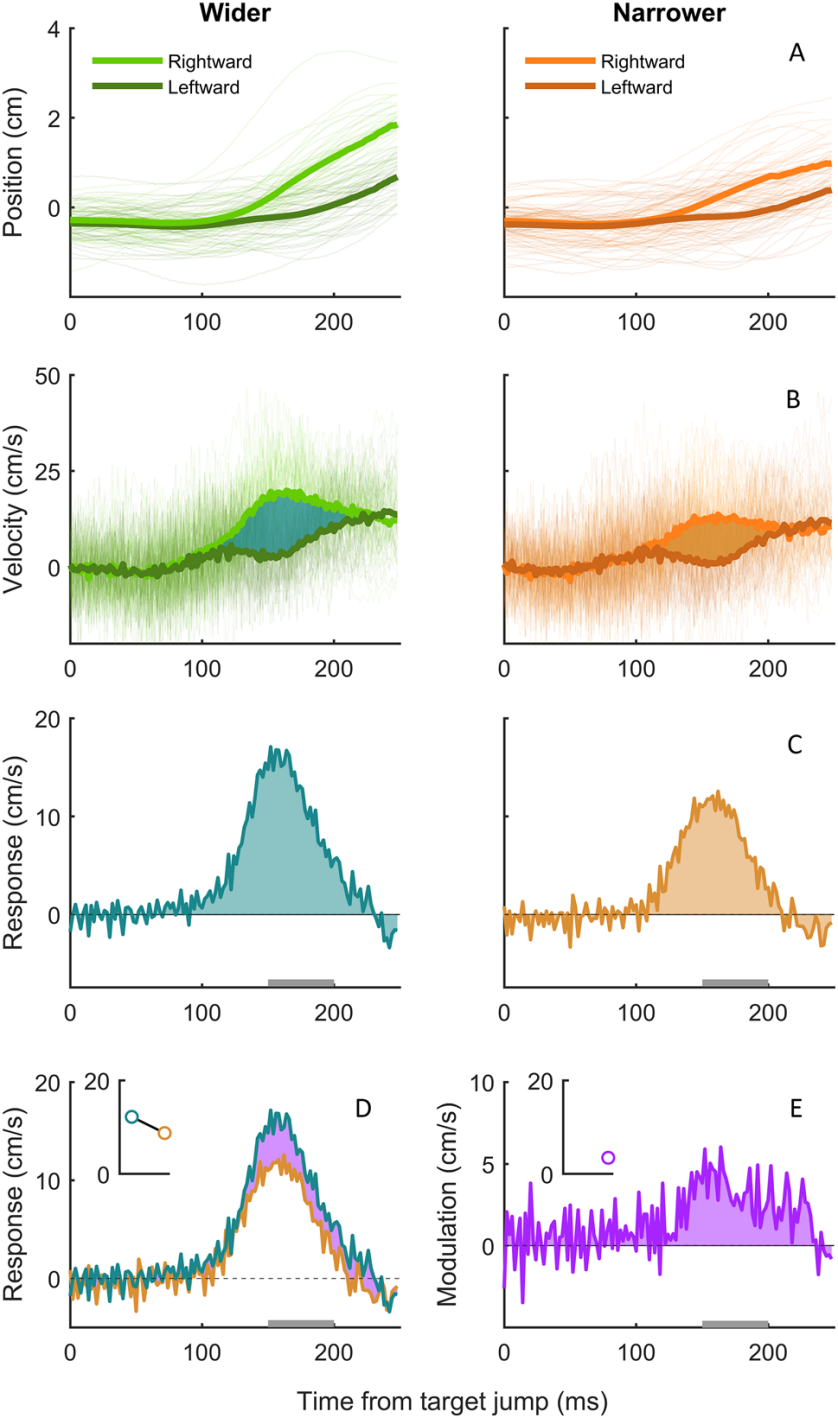
Overview of the steps in data analysis using data for the late conditions from one participant in the obstacles group. **A** Position and **B** velocity of the finger as a function of time since the target jump. Thin lines show individual trials; thick lines show the mean. Left and right panels of the top three rows show the conditions where the gap size widened and narrowed, respectively. Colour indicates the direction of the jump. **C** The response to the target jump (shaded areas in **B**), calculated by subtracting the average velocity after leftward jumps from that after rightward jumps at each time. Positive values indicate that there was a response in the direction of the target jump. **D** The two response curves (from the two panels of **C**) plotted together to illustrate the difference between them (shaded area; response modulation). **E** The response modulation, calculated at each time from the target jump by subtracting the response when the gap size narrowed from that when it widened. The formatting of panels **D** and **E** will be used in the results section

All trials were included in the analysis, irrespective of performance. To evaluate the time course of how participants responded to the lateral target jump, we analysed the lateral motion of the finger. We first converted the measured lateral positions of the finger (Fig. 3a) into lateral velocities (Fig. 3b). This was done for every 2 ms interval for the first 250 ms after the target jumped (300–550 ms after the target appeared). We chose to plot the response until 550 ms because, on average, this captures the finger movements up until just after the point of interception. For each participant, we then averaged the lateral velocity of the finger for every interval. We did so separately for trials in which the target jumped leftward and ones in which it jumped rightward. We subsequently determined the *response* to the target jump by subtracting the average velocity after leftward jumps from that after rightward jumps (Fig. 3c, d). We did this for each timing of item movement (early and late) and change in gap size (wider or narrower).

To assess whether the response to the target jump was modulated by the change in gap size, we determined the *response modulation* by subtracting the response (difference in average velocity after leftward and rightward jumps) when the gap size narrowed from that when it widened (Fig. 3e). We did this for each timing of item movement (early and late). For the statistical analysis, we quantified the *response modulation magnitude* for each participant by taking the mean response modulation between 150 and 200 ms after the target jumped. We chose this time window because the exact timing of the peak of the response depends on the remaining time in the movement, but it always falls within this interval (Brenner et al. 2022). After determining the individual responses and response modulations in each condition, the values were averaged across participants.

To determine whether the response to the target jump was modulated by the change in gap size when the obstacles changed position at the same moment that the target jumped (late), we used a one-tailed, one-sample *t*-test to evaluate whether the response modulation magnitude was significantly larger than zero. Since it was, we needed to determine whether this is really due to the continuous updating of obstacle positions, rather than being caused by the obstacles’ motion itself. This was done by evaluating whether the response modulation magnitude was larger for the late condition of the obstacle than for the late condition of the irrelevant item group (using a one-tailed, two-sample *t*-test). If the response modulation magnitude in the late obstacles group had not been significantly larger than zero, we would not have conducted this test, but we would have checked that the response to the target jump did differ if the gap size was known in advance. This would have been done by evaluating the response modulation magnitude for the early gap changes for the obstacle group (again using a one-tailed, one-sample *t*-test).

## Results

Table 1 gives an overview (mean ± standard deviation) of the number of targets and red items hit in each condition, split according to the group. Participants in the irrelevant group hit the target more often than those in the obstacles and replication group, presumably because adjustments to their movements were not constrained by the obstacles, and because they could hit the target later and further to the right. Figure 2 confirms that the fingers of the irrelevant group moved further to the right. The target hit rate was similar across all conditions in the irrelevant group, in accordance with participants ignoring the irrelevant items such that the timing of their movement and the change in gap size had no effect on performance. In contrast, participants in the obstacles and replication group tended to perform worse when the gap narrowed compared to when it widened. Participants in the irrelevant group passed through the irrelevant items considerably more than participants in the obstacles and replication groups passed through the obstacles. All these findings indicate that all groups adhered to their instructions. In all groups, there were collisions with more items when the gap narrowed. Overall, Table 1 shows that the obstacles were effective: items were clearly avoided by the groups that were told that the items were obstacles, at the expense of missing the target more often.

**Table 1:** Overview of the percentage (mean ± standard deviation) of trials on which the target and red items were hit in each condition, split according to the different groups

Group	Timing of item movement	Gap size change	Target jump direction	Items hit (%)		
				Targets	Right item	Left item
Obstacle	Early	Wider	Left	87 ± 6	0 ± 1	0 ± 0
			Right	84 ± 9	0 ± 1	0 ± 0
		Narrower	Left	82 ± 11	3 ± 4	8 ± 7
			Right	62 ± 17	10 ± 8	4 ± 4
	Late	Wider	Left	85 ± 9	0 ± 0	0 ± 1
			Right	78 ± 12	0 ± 0	0 ± 0
		Narrower	Left	76 ± 14	4 ± 5	10 ± 9
			Right	64 ± 19	11 ± 8	5 ± 5
Irrelevant	Early	Wider	Left	87 ± 7	3 ± 7	0 ± 0
			Right	90 ± 6	4 ± 7	0 ± 0
		Narrower	Left	85 ± 9	25 ± 25	0 ± 1
			Right	87 ± 7	42 ± 27	0 ± 0
	Late	Wider	Left	85 ± 9	3 ± 5	0 ± 0
			Right	88 ± 6	5 ± 9	0 ± 0
		Narrower	Left	89 ± 7	31 ± 28	0 ± 1
			Right	86 ± 9	44 ± 33	1 ± 0
Replication	Early	Wider	Left	80 ± 14	0 ± 1	0 ± 0
			Right	77 ± 16	0 ± 0	0 ± 0
		Narrower	Left	74 ± 21	3 ± 4	5 ± 3
			Right	59 ± 21	10 ± 8	3 ± 5
	Late	Wider	Left	80 ± 16	0 ± 1	0 ± 0
			Right	72 ± 21	0 ± 0	0 ± 0
		Narrower	Left	74 ± 22	3 ± 3	7 ± 6
			Right	60 ± 22	8 ± 7	4 ± 4

**Fig. 4:**
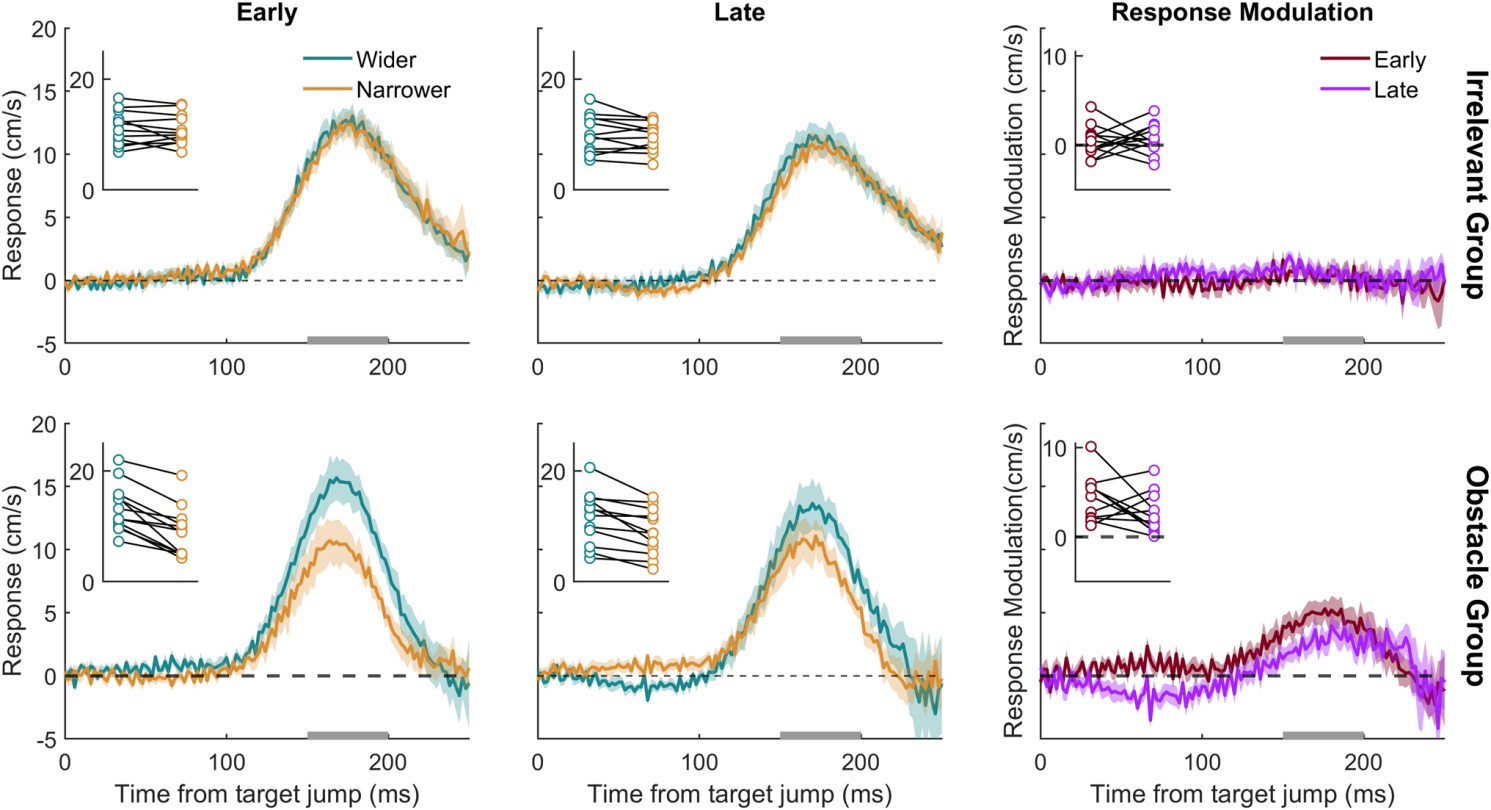
Time course of the hand’s response to target jumps in the main experiment. Positive values indicate that the response is in the direction of the target jump. The rows correspond to the two groups that participants were assigned to; the left and central columns correspond to the two timings of item movement; the curve colours correspond to the change in gap size and match the frames in Fig. 1. Curves correspond to the mean values. Shaded areas show the standard errors across participants. The time course of the response modulation (difference between gold and turquoise curves) for the two groups of participants is shown in the right panels. Curves deviating from zero indicate that the response to the target jump was modulated by the change in gap size. Positive values indicate that the response was larger when the gap size widened; negative values indicate that the response was larger when the gap size narrowed. The curve colours correspond to the timing of item movement. The inset in the top left of each figure shows the mean response (left and central panels) or response modulation (right panel) between 150 and 200 ms from the target jump for each participant. This interval is denoted by the grey rectangle on the *x*-axis*.* Each participant’s data points are connected by lines

As was to be expected, there was a clear response of the hand to the target jump in all conditions, demonstrated by the response curves all clearly deviating from zero in the positive direction. The hand moved in the direction of the target jump (positive values in left and central panels of Fig. 4). The response to the target jump was not modulated by the final gap size in the irrelevant item group, evidenced by the responses being similar for wider and narrower gaps (gold and turquoise curves in upper panels of Fig. 4), and consequently the response modulation not deviating from zero (red and purple curves in upper right panel of Fig. 4). The response to the target jump was modulated by the gap size in the obstacles group, evidenced by the responses being stronger when the gaps became wider than when they became narrower (turquoise curves above gold curves in lower panels of Fig. 4; red and purple curves systematically above zero at the time of the response). This was the case for every participant (turquoise points above the associated gold points and all red and purple points above zero in the insets of the same panels), so not surprisingly the response modulation for the critical, late condition (purple curve in bottom right panel of Fig. 4) was significantly larger than zero *t*(11) = 4.00, *p* = 0.001. The response modulation was also significantly larger for late changes in the obstacles’ positions than for late changes in the irrelevant items’ positions (*t*(22) = 2.36, *p* = 0.014; purple curves in Fig. 4).

Surprisingly, the response modulation curve for the late changes in the obstacles group is below zero between about 25 and 100 ms after the jump (purple curve in lower right panel of Fig. 4). Adjustments to a target jump are typically observed from about 100 ms after the jump at the earliest (e.g. Brenner and Smeets 1997; Day and Lyon 2000; Prablanc and Martin 1992; Smeets et al. 1998; Soechting and Lacquantiti 1983). Thus, much earlier differences cannot be attributed to the target jump, so we regard this to be a coincidence. To verify this interpretation of the data of the critical condition of the obstacles group (that the modulation before 100 ms was a coincidence, but the later modulation was not), we replicated the experiment for the obstacles group.

## Replication Experiment

This experiment was a direct replication of the *obstacles* group of the main experiment, using the same methods. The only difference was that the replication group consisted of 12 new participants who took part in the experiment in return for course credit (10 right-handed; 21 ± 2 years).

## Results

**Fig. 5:**
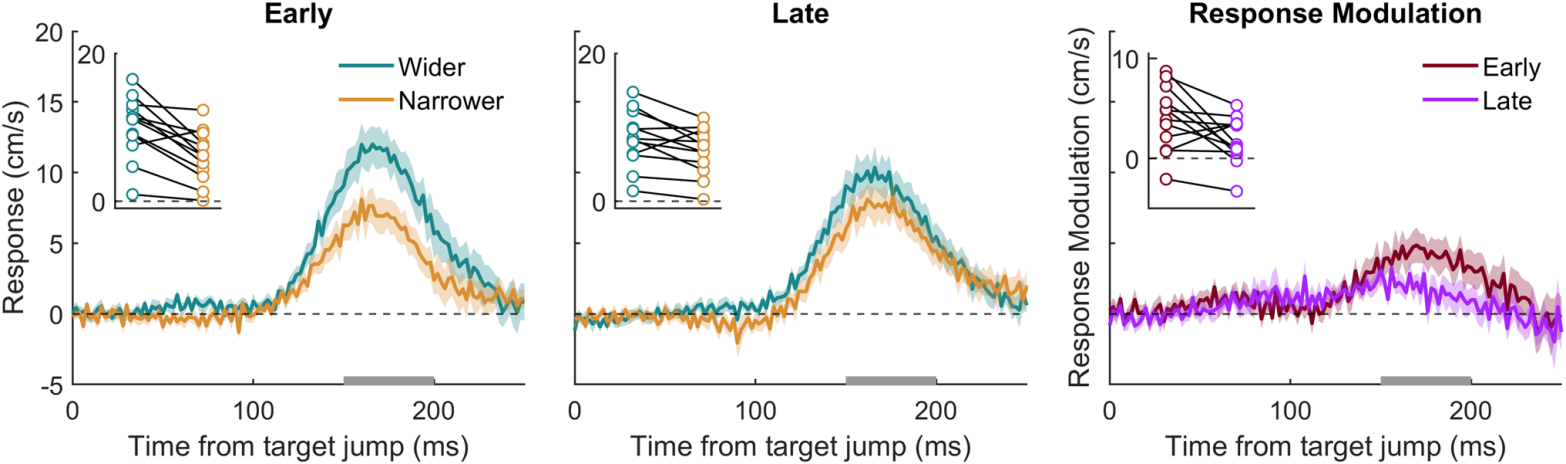
Time course of the hand’s response to target jumps for the four conditions in the replication experiment (left and central panel). Time course of the response modulation (right panel). The inset in the top left of each figure shows the mean response (left and central panels) or response modulation (right panel) between 150 and 200 ms from the target jump for each participant of the replication group. This interval is denoted by the grey rectangle on the *x*-axis*.* This experiment is a replication of the obstacles group presented in the lower row of Fig. 4. Further details as in Fig. 4

The overall pattern of results is consistent with the main experiment: participants hit less targets and collided with more obstacles when the gap narrowed compared with when it widened (Table 1). Participants did, however, tend to hit fewer targets and also fewer obstacles in the replication experiment. This suggests that they adjusted their movements less vigorously, which will reduce the ability to hit the target but also reduce the likelihood of hitting obstacles. Indeed, Fig. 5 shows that participants adjusted less vigorously than in the main experiment (Fig. 4).

Again, there was a clear response of the hand to the target jump in all four conditions (left and central panels of Fig. 5), and the response to the late change in gap size was smaller when the gap became narrower than when the gap widened (response modulation larger than zero between 150 and 200 ms after the target jump; *t*(11) = 2.60, *p* = 0.013). In the replication, two of the participants did not have a positive response modulation in the late condition, but the one that clearly responded more vigorously when the obstacles moved closer together also did so in the early condition. We have no explanation for this participant’s behaviour. Importantly, the response modulation effect between 150 and 200 ms after the target jump was replicated. In contrast, the early negative response modulation (before 100 ms) was not, supporting our interpretation that it was a coincidence.^1^

## Discussion

Our goal was to assess whether the positions of obstacles in the surrounding are constantly updated when moving to intercept a target. To do this, we designed a task in which participants had to respond to a sudden change in the target’s position and assessed whether the size of this response was dependent upon the gap size between two obstacles through which participants must pass. In the critical condition, the gap size changed at the same time as the target jumped. The response to the target jump was modulated by the size of the gap between the obstacles: the magnitude of the response to the target jump was smaller when the gap between the obstacles was smaller. This provides evidence that obstacle positions can be updated and incorporated into the adjustment of an ongoing reaching movement.

Before discussing this interpretation further, we should rule out alternative explanations. The first is that the modulation of responses by changes in gap size have nothing to do with considering the latest positions of the obstacles, but are automatic responses to visual motion, as was suggested by Aivar et al. (2015). Such automatic responses are frequently observed when irrelevant items move in the background (e.g. Brenner and Smeets 1997, Saijo et al. 2005; Crowe et al. 2021). To rule out this explanation, we included an irrelevant items group in the main experiment. The participants in this group viewed exactly the same stimulus as the other group but were told that the red items were irrelevant to the task. Accordingly, there was no penalty if they were hit. For the participants in this group, the change in the position of the irrelevant items did not influence the response (response modulation curve does not deviate systematically from zero in Fig. 4), in contrast to the participants who were told that the red items were obstacles. Therefore, automatic responses to visual motion cannot underlie the modulation of the response in the obstacles group.

We designed the study in a way that balanced the motion of nearby items to minimise the potential effect of any automatic responses to background motion. However, the finger moved further to the right when the red items were irrelevant, rather than moving straight between the items (Fig. 2), and consequently hit the right irrelevant items on 20% of the trials, whereas it only hit the left irrelevant items on less than 1% of trials and only hit the right items when they were obstacles on 3% of the trials. The fact that the finger moved further to the right disturbed the balancing that we hoped to achieve by having the two items move in opposite directions at equal distances from the finger for irrelevant items. The finger might therefore have moved rightwards automatically when the items moved apart in the late condition, because the closest, rightmost item moved to the right, and automatically moved leftward when the items moved closer together, because the rightmost item moved to the left (Brenner and Smeets 2015; Crowe et al. 2021, 2022). But this should not influence our analysis, as long as such additional lateral motion does not influence the response to the target jumps, because the response is the difference between how the trajectory changes after a leftward and rightward target jump. Indeed, no difference was found. What we do find, which was the main purpose of the study, is that the response to target jumps is not so automatic that obstacle positions are ignored (as already demonstrated by Chapman and Goodale 2010b and Nashed et al. 2012 for obstacles that were present from movement onset). It is possible that the response modulation found in our experiments was actually also present in the studies by Aivar et al. (2008, 2015), but that it was masked by a stronger automatic response to the visual motion.

In the early conditions, when the gap changed at the time of the target appearance, the obstacle positions could be incorporated into the initial movement plan. The fact that the response to the target jump considered the position of the obstacles in the early condition is in line with an earlier study showing that obstacles affect arm movement adjustments (Chapman and Goodale 2010b; Nashed et al. 2012). Our new finding is that when the gap changed size at the time of the target jump (late), the response to the target jump was still modulated by the new position of the obstacles (Figs. 4, 5). This shows that ongoing movements do not only consider the most recent target position, but also the most recent obstacle positions. In this experiment, participants could have tuned into the fact that there would always be a change in gap size, although they could not know the direction. It is therefore possible that the response modulation is particularly large in our experiment because participants could anticipate a perturbation. We cannot be sure that participants would respond in the same way if the gap size only changed on a small percentage of trials, but participants must respond quickly to the target jump itself such that the response is unlikely to be controllable (Prablanc and Martin 1992; Brenner et al. 2022a, b).

The response modulation revealed in the obstacles group appears to be stronger when the gap changed size earlier. This is not the result of moving in a manner that increases the possible vigour when one knows the gap size in advance. We interleaved all the conditions, but participants could have responded to early motion of the obstacles by adjusting how they moved towards the target. However, it is evident from Fig. 2 that the finger moved in a very similar manner in the early and late conditions until the response started: 100 ms after the target jump. Thus, the timing as well as the direction of the obstacle displacements influenced how the finger reacted to the target jumps, but the movement was not different before the response.

Here, we perturbed the target to enable us to assess whether the response to such a perturbation was modulated by the position of the obstacles. Other research has used mechanical perturbations whereby the arm is perturbed by applying a mechanical force to assess the effect of obstacles on adjustments to the ongoing movements. De Comite et al. (2021) showed that the response to a mechanical perturbation was modulated by the sudden appearance or disappearance of obstacles in the surrounding. In our experiment, the obstacles were always present from the onset of the trial and jumped in unpredictable directions. In contrast, De Comite et al. (2021) always had the obstacles either appear or disappear at a fixed location such that participants could tune into the way in which the environment could change. Nevertheless, their results and interpretation fit with our conclusion that the instantaneous positions of obstacles can be incorporated into the adjustment of an ongoing goal-directed movement.

## Conclusion

To successfully execute reaching movements, people continuously update the position of both their hand and of the target item they wish to interact with. Using an interception task with virtual objects, we here show that people can also continuously update the positions of obstacles in the surrounding when adjusting their movements. The short latency with which all this takes place provides evidence for a highly automatic system that uses the most recent information about both target and obstacle positions to guide ongoing arm movements. This presumably contributes to the fact that people rarely collide with the many obstacles that they encounter in their surroundings in daily life.

## Data Availability

The data and analysis scripts for this study are available at: https://osf.io/4fjru/?view_only=.

## Footnotes

1 One of the reviewers requested consideration of a wider time-window (100–200 ms). Doing so did not change the conclusions: the response modulation by the obstacles in the late condition was significantly greater than zero in both the main experiment (*t*(11) = 4.40, *p* < 0.001) and the replication (*t*(11) = 2.56, *p* = 0.013).
